# High Pressure‐Driven Magnetic Disorder and Structural Transformation in Fe_3_GeTe_2_: Emergence of a Magnetic Quantum Critical Point

**DOI:** 10.1002/advs.202206842

**Published:** 2023-01-25

**Authors:** Ngoc‐Toan Dang, Denis P. Kozlenko, Olga N. Lis, Sergey E. Kichanov, Yevgenii V. Lukin, Natalia O. Golosova, Boris N. Savenko, Dinh‐Loc Duong, The‐Long Phan, Tuan‐Anh Tran, Manh‐Huong Phan

**Affiliations:** ^1^ Institute of Research and Development Duy Tan University Da Nang 550000 Vietnam; ^2^ Faculty of Environmental and Natural Sciences Duy Tan University Da Nang 550000 Vietnam; ^3^ Frank Laboratory of Neutron Physics JINR Moscow Reg. Dubna 141980 Russia; ^4^ Kazan Federal University Kazan 420008 Russia; ^5^ Center for Integrated Nanostructure Physics Institute for Basic Science Suwon 16419 Republic of Korea; ^6^ Faculty of Engineering Physics and Nanotechnology VNU‐University of Engineering and Technology 144 Xuan Thuy, Cau Giay Ha Noi 100000 Vietnam; ^7^ Ho Chi Minh City University of Technology and Education Ho Chi Minh 700000 Vietnam; ^8^ Department of Physics University of South Florida Tampa FL 33620 USA

**Keywords:** high pressure, magnetic quantum criticality, vdW magnets

## Abstract

Among the recently discovered 2D intrinsic van der Waals (vdW) magnets, Fe_3_GeTe_2_ (FGT) has emerged as a strong candidate for spintronics applications, due to its high Curie temperature (130 – 220 K) and magnetic tunability in response to external stimuli (electrical field, light, strain). Theory predicts that the magnetism of FGT can be significantly modulated by an external strain. However, experimental evidence is needed to validate this prediction and understand the underlying mechanism of strain‐mediated vdW magnetism in this system. Here, the effects of pressure (0 – 20 GPa) are elucidated on the magnetic and structural properties of Fe_3_GeTe_2_ by means of synchrotron Mössbauer source spectroscopy, X‐ray powder diffraction and Raman spectroscopy over a wide temperature range of 10 – 290 K. A strong suppression of ferromagnetic ordering is observed with increasing pressure, and a paramagnetic ground state emerges when pressure exceeds a critical value, *P*_PM_ ≈ 15 GPa. The anomalous pressure dependence of structural parameters and vibrational modes is observed at *P*_C_ ≈ 7 GPa and attributed to an isostructural phase transformation. Density functional theory calculations complement these experimental findings. This study highlights pressure as a driving force for magnetic quantum criticality in layered vdW magnetic systems.

## Graphical abstract

Strong magnetic ordering suppression is discovered under high pressure, and a paramagnetic ground state emerges when pressure exceeds ≈15 GPa in Fe_3_GeTe_2_. An isostructural phase transformation occurs at ≈7 GPa. These findings highlight pressure as a driving force for magnetic quantum criticality in layered van der Waals (vdW) magnets like Fe_3_GeTe_2_, rather than chemical doping that often induces structural disorder.

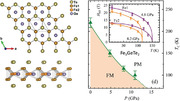

## 1 Introduction

Following the discovery of graphene – the strongest and thinnest 2D van der Waals (vdW) material, numerous efforts have been made to search for new 2D vdW materials with outstanding optoelectronic and magneto‐optical properties for ultrathin, ultracompact, and low‐power nanodevice applications.^[^ ^1^, ^2^, ^3^ ^]^ The recent discovery of ferromagnetism in 2D vdW magnetic systems, such as CrI_3_,^[^ ^4^ ^]^ Cr_2_Ge_2_Te_2_,^[^ ^5^ ^]^ Fe_3_GeTe_2_,^[^ ^6^ ^]^ Fe_5_GeTe_2_,^[^ ^7^ ^]^ VSe_2_,^[^ ^8^ ^]^ MnSe_2_,^[^ ^9^ ^]^ CrSe_2_,^[^ ^10^ ^]^ CrTe_2_,^[^ ^11^ ^]^ and *TX*_2_ (*T* = W, Mo; *X* = V, Fe)^[^ ^12^, ^13^, ^14^, ^15^ ^]^ has provided new, unprecedented opportunities for control and manipulation of magnetism and spin transport phenomena down to the monolayer limit. Unlike conventional 3D magnetic systems, these 2D vdW magnets exhibit strong magnetic responses to external stimuli (magnetic field, electric field, light, strain),^[^ ^16^, ^17^, ^18^, ^19^, ^20^ ^]^ making them ideal candidates for wide ranging applications in spintronics, opto‐spintronics, straintronics, opto‐spin‐caloritronics, and computation.^[^ ^2^, ^3^, ^21^ ^]^

**Figure 1:**
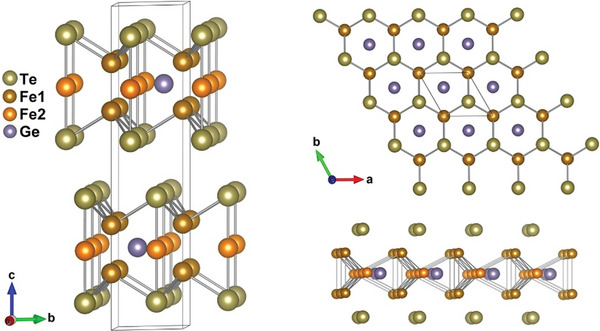
The hexagonal crystal structure of Fe_3_GeTe_2_. The unit cell, top view, and side view of a single vdW layer of Fe_3_GeTe_2_ are shown.

Among cleavable layered 2D vdW magnetic materials, Fe_3_GeTe_2_ (FGT) is of particular interest since it demonstrates itinerant ferromagnetism (FM) with a high Curie temperature of *T*_C_ ≈ 220 K in its bulk stoichiometric form^[^ ^22^ ^]^ and exhibits new, exotic physics down to the atomically thin level.^[^ ^2^, ^16^, ^17^ ^]^ In the monolayer limit, the *T*_C_ value is significantly reduced to ≈130 K, but can be boosted to room temperature by ionic gating.^[^ ^6^, ^20^ ^]^ FGT crystallizes in a hexagonal structure with *P6_3_/mmc* symmetry, where Fe_3_Ge layers are separated by two Te layers, coupled by vdW bonds (**Figure** **1**). The ferromagnetism of FGT is derived from two inequivalent Fe1^3+^ and Fe2^2+^ sites within the monolayer. It has been reported that bulk FGT exhibits a large out‐of‐plane magnetic anisotropy with respect to the vdW planes, and the strength of this anisotropy remains significant in the monolayer, thus preserving the long‐range FM order.^[^ ^6^ ^]^ FGT demonstrates extreme sensitivity to the change of thermodynamic parameters (external magnetic and electric fields, chemical and electronic doping, strain), leading to the appearance of emergent phenomena like tunneling spin‐valve behavior, the anomalous Hall effect, heavy fermion states, chiral spin structures including skyrmions and spin spirals, etc.^[^ ^23^, ^24^, ^25^, ^26^, ^27^ ^]^ FGT is also a topological FM nodal line semimetal.^[^ ^26^, ^28^ ^]^

The low‐dimensional nature of FGT's structure facilitates pronounced quantum fluctuations at certain physical conditions, so the application of an external stimulus such as pressure may drive the system into a quantum critical regime.^[^ ^29^ ^]^ The application of pressure has been demonstrated to be an effective approach for manipulating the magnetic properties of materials without changing chemical compositions.^[^ ^30^, ^31^, ^32^, ^33^ ^]^ Theory predicts that the Curie temperature and coercivity of FGT can be increased by an applied tensile strain,^[^ ^34^ ^]^ which has been confirmed experimentally.^[^ ^19^ ^]^ Interestingly, Hall measurements performed by Wang et al.^[^ ^19^ ^]^ showed that the application of a small uniaxial tensile strain (≈0.32) to FGT nanoflakes increased the coercive field by 150%. However, the strong suppression of the ferromagnetism and Curie temperature of FGT upon application of high pressures (compressive strain) were reported.^[^ ^35^, ^36^, ^37^, ^38^ ^]^ This was attributed to decreases in the exchange interaction and the magnetocrystalline anisotropy,^[^ ^35^, ^37^ ^]^ however, a clear correlation between the structure and magnetism was not established. Previous study revealed the pressure‐modulated magnetism in FGT in a limited pressure range (0–12 GPa), within which a complete suppression of the FM ordering was not reached.^[^ ^37^ ^]^ Therefore, it remains unknown at which pressure (considered as a critical pressure) the FM ordering is fully suppressed, and what will govern the magnetic ground state when pressure exceeds this critical value. While Wang et al.^[^ ^38^ ^]^ and O'Hara et al.^[^ ^36^ ^]^ observed a monotonic decrease in *T_C_* with increasing pressure up to 12 GPa, with no evidence of structural change in FGT up to 29.4 GPa, a significant change in shape of the magnetic hysteresis loop at *P* ≈ 7 GPa was observed in FGT by magnetic circular dichroism spectroscopy (MCDS) and was suggested to arise from a pressure‐induced structural phase transformation (e.g., the change in the Fe1‐Te bond length) by density functional theory (DFT) calculations.^[^ ^37^ ^]^ Such a discrepancy among these works^[^ ^36^, ^37^, ^38^ ^]^ may arise from the fact that the pressure‐induced structural change at *P* ≈ 7 GPa could be relatively weak and less pronounced in defective Fe_3‐x_GeTe_2_ crystals.^[^ ^36^ ^]^ In the latter case,^[^ ^37^ ^]^ no pressure‐related structural characterization experiment was performed, so correlation between the structural and magnetic changes ≈7 GPa was not established and remained an open question.

To shed light on these important, unsolved issues and possibly find a pressure‐driven magnetic quantum critical point (MQCP) in a layered vdW magnet like FGT, we have performed a comprehensive study of the magnetic and structural properties of single crystalline FGT in the powder form by means of synchrotron Mössbauer spectroscopy (SMS), X‐ray powder diffraction (XRD), and Raman spectroscopy (RS) measurements over the 0 – 20 GPa pressure and 10 – 300 K temperature ranges. The SMS results show a strong suppression of hyperfine magnetic fields and ferromagnetic ordering with increasing pressure, and a paramagnetic ground state emerges when pressure exceeds a critical value, *P*_PM_ ≈ 15 GPa. The analyses of the XRD and RS results provide the first experimental evidence for the anomalous pressure dependence of structural parameters and vibrational modes at *P*_C_ ≈ 7 GPa and have attributed this to the isostructural phase transformation, which are complemented by DFT calculations. These experimental observations also validate the previous DFT‐based prediction^[^ ^37^ ^]^ and allow us to establish, for the first time, the correlation between the structural and magnetic changes ≈7 GPa. Our study also highlights pressure as a potential driving force for magnetic quantum criticality in layered vdW magnetic systems like FGT, rather than chemical doping approaches that may induce structural disorder.^[^ ^39^, ^40^ ^]^

## 2 Results and Discussion

### 2.1 Synchrotron Mössbauer Source Spectroscopy

**Figure 2:**
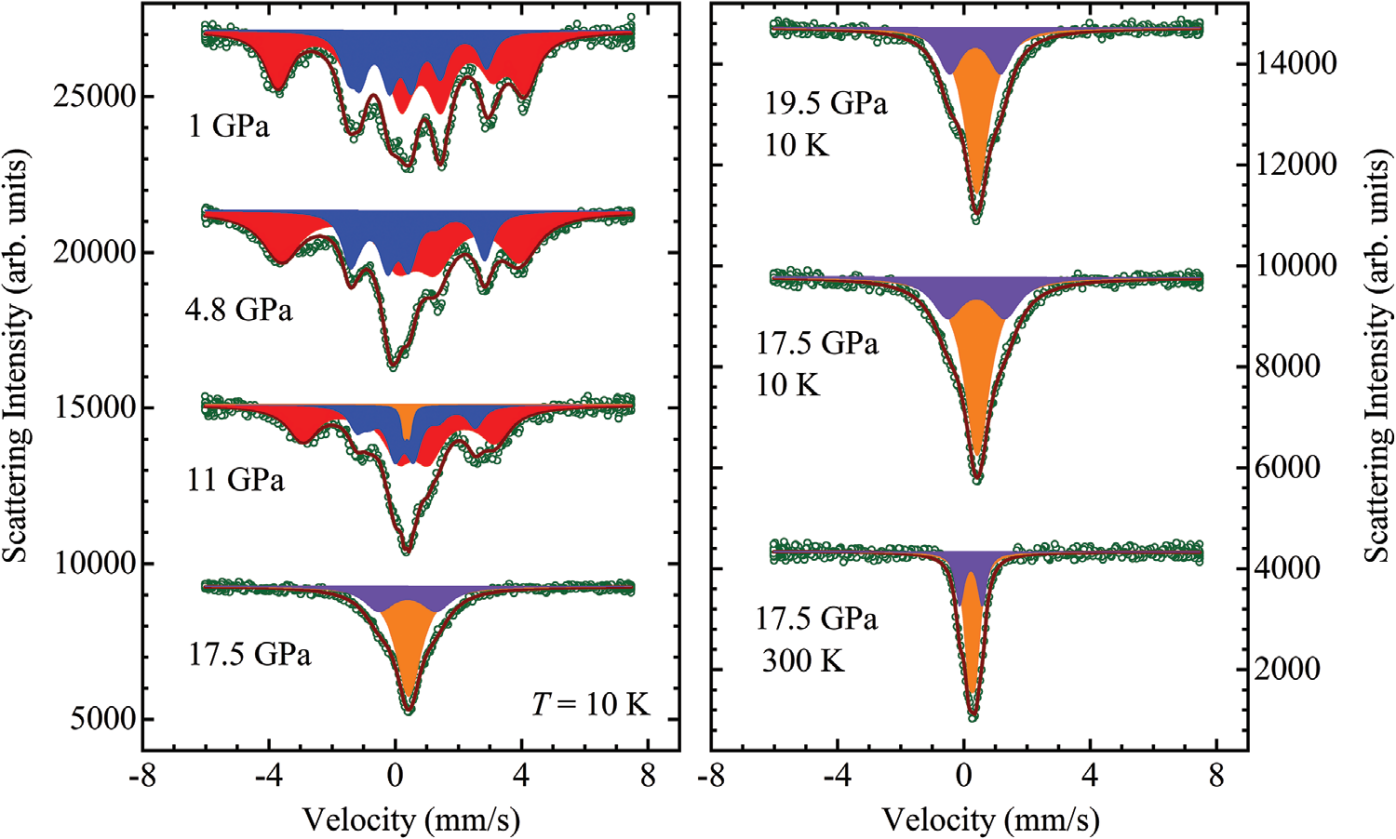
The SMS spectra of Fe_3_GeTe_2_, measured at selected pressures and temperatures. The experimental points (symbols) and fitting curves (solid lines) are shown. The sextet components in the ferromagnetically ordered phase corresponding to two nonequivalent Fe sites are shown in red and blue colors. The doublet components in the pressure‐induced paramagnetic phase corresponding to two nonequivalent Fe sites are shown in orange and violet colors.

The synchrotron Mössbauer spectra of Fe_3_GeTe_2_ under various pressures measured at low (e.g., *T* = 10 K) and high (e.g., *T* = 300 K) temperatures are shown in **Figure** **2**.

It can be seen that below the Curie temperature (≈220 K), for pressures up to 11 GPa, the spectra measured at 10 K are fitted well to the model containing two sextet components, corresponding to the Fe1 (4*e*) and Fe2 (2*c*) inequivalent sites in the hexagonal structure of *P6_3_/mmc* symmetry. The intensity ratio of the relevant sextets was fixed to 2:1 according to the site occupancies during the fitting procedure. For *P* = 1 GPa and *T* = 10 K, the obtained hyperfine parameters values, including isomer shifts *IS*_1_ = 0.50(5) and *IS*_2_ = 0.43(3) mm/s, quadrupole splittings *QS*_1_ = ‐0.63(3) and *QS*_2_ = 0.53(4) mm/s, and hyperfine magnetic field *H*_hf1_ = 24.2(2) T, are comparable with those reported for ambient pressure at *T* = 77 K.^[^ ^41^ ^]^ However, the determined hyperfine field *H*_hf2_ = 13.6(2) T for the Fe2 (2*c*) sites is greater than that of 5.6 T reported in.^[^ ^41^ ^]^ The latter value seems to be severely underestimated, since from the ordered magnetic moments *m*_1_ = 2.18 and *m*_2_ = 1.54 µ_B_ for the Fe1 and Fe2 sites^[^ ^42^ ^]^ one can estimate the expected hyperfine magnetic fields ratio *H*_hf1_/*H*_hf2_ ≈ 1.42 according to the known linear scaling between *H*_hf_ and *m* quantities.^[^ ^43^ ^]^ Our experimental value, *H*_hf1_/*H*_hf2_ = 1.78, is comparable with the estimated one, and the slight difference may be related to some variation of Fe stoichiometry in the presently studied FGT.^[^ ^41^ ^]^

**Figure 3:**
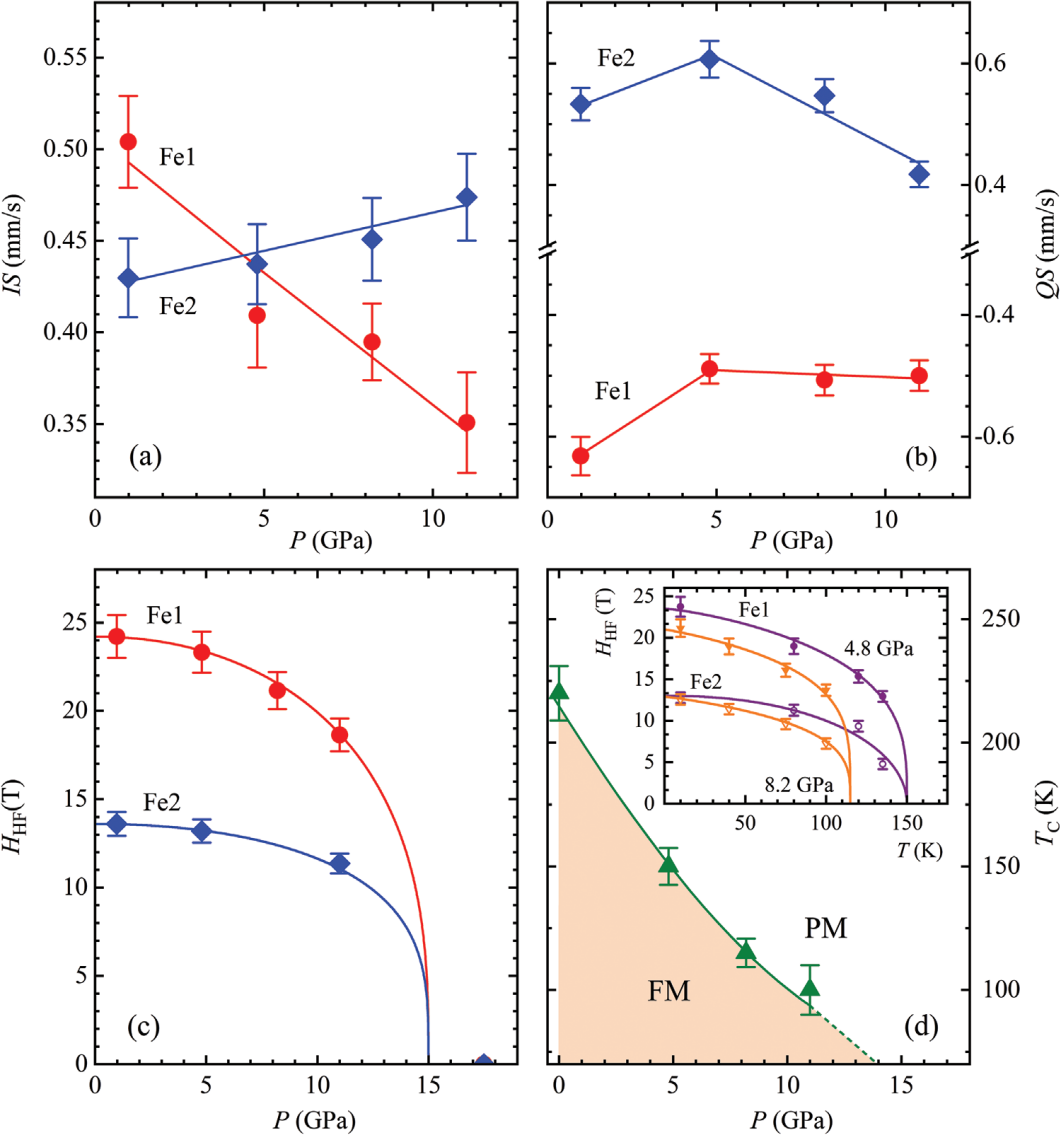
The hyperfine parameters (*IS*, *QS*, *H*_HF_) of Fe_3_GeTe_2_ as functions of pressure at *T* = 10 K a–c). The red circles and blue rhombi correspond to Fe1 and Fe2 sites. The bars represent computational errors obtained by the Moussa program. The pressure dependence of the Curie temperature (d). The line is a guide to eyes only. Temperature dependences of hyperfine magnetic fields at *P* = 4.8 and 8.2 GPa, fitted to Equation: *H*_HF_(*T*)  =  *H*_HF0_(1‐(*T*/*T*_C_)^*α*^)^*β*^ (inset).

Upon lattice compression at *T* = 10 K, the Fe1 isomer shift (IS) demonstrates a nearly linear reduction, while the Fe2 IS follows an opposite trend with less pronounced variation (**Figure** **3**). The quadrupole shifts (QS) show an anomalous pressure behavior. Both QS values for Fe1 and Fe2 sites follow an increasing trend below *P* ≈ 5 GPa. At higher pressures, however, the QS value of Fe1 sites decreases rapidly, while those of the Fe2 sites remain nearly unchanged. For pressures up to 11 GPa, the hyperfine magnetic fields of the Fe1 and Fe2 sites are reduced progressively by 23 and 16%, respectively. We evaluate a critical pressure *P*_PM_ ≈ 15 Gpa for a full suppression of the FM state in Fe_3_GeTe_2_ from the fit of the pressure dependences of the hyperfine fields by an empirical function *H*_HF_(*P*)  =  *H*_HF0_(1‐(*P*/*P*_C_)^*α*^)^*β*^. The Curie temperature decreases from *T*_C_ ≈ 220(5) K at ambient pressure to 100(5) K at 11 Gpa, as evaluated from the temperature dependences of hyperfine magnetic fields (Figure 3). The obtained value of *T*_C_ ≈ 220 K at *P* = 0 Gpa is consistent with that obtained from magnetometry. The relevant pressure coefficient of *dT_C_/dP* = ‐11 K/Gpa is comparable with previous estimations which ranged from ‐13 K/GPa to ‐7.4 K/GPa, which were based on transport and magnetization measurements.^[^ ^36^, ^38^, ^44^ ^]^

At *P* = 11 GPa and low temperatures (10 K), additional paramagnetic doublet components appeared (Figure 2). At *P* = 17 GPa, however, only paramagnetic doublets were detected in the SMS spectra, while sextet components corresponding to the ferromagnetically ordered state were fully suppressed (Figure 2). The relevant hyperfine parameters are *IS* = 0.42(1) mm/s, *QS* ≈ 0.00(1) mm/s for the Fe1 sites and *IS* = 0.39(2) mm/s, *QS* ≈ 1.81(5) mm/s for the Fe2 sites. With a pressure increase up to 19.5 GPa, these values change slightly to *IS* = 0.41(1) mm/s, *QS* ≈ 0.00(1) mm/s for the Fe1 sites and *IS* = 0.36(2) mm/s, *QS* ≈ 1.62(4) mm/s for the Fe2 sites.

The gradual suppression of the FM state in Fe_3_GeTe_2_ upon compression towards *P*_PM_ = 15 GPa, corresponds to a full vanishing of the hyperfine magnetic fields (Figure 3). Further, *T*_C_ = 0 K, implies that Fe_3_GeTe_2_ is a promising candidate material in the search for a pressure‐induced ferromagnetic quantum critical point (QPT). Further confirmation of the QPT existence would require precise structural and magnetic measurements near *T*_C_ = 0 K at high pressures. This would confirm the second order character of the phase transition to a paramagnetic state,^[^ ^45^ ^]^ which is not possible within our experimental setup due to challenges controlling high pressures in the diamond anvil cell at temperatures below 10 K.

### 2.2 X‐Ray Diffraction

**Figure 4:**
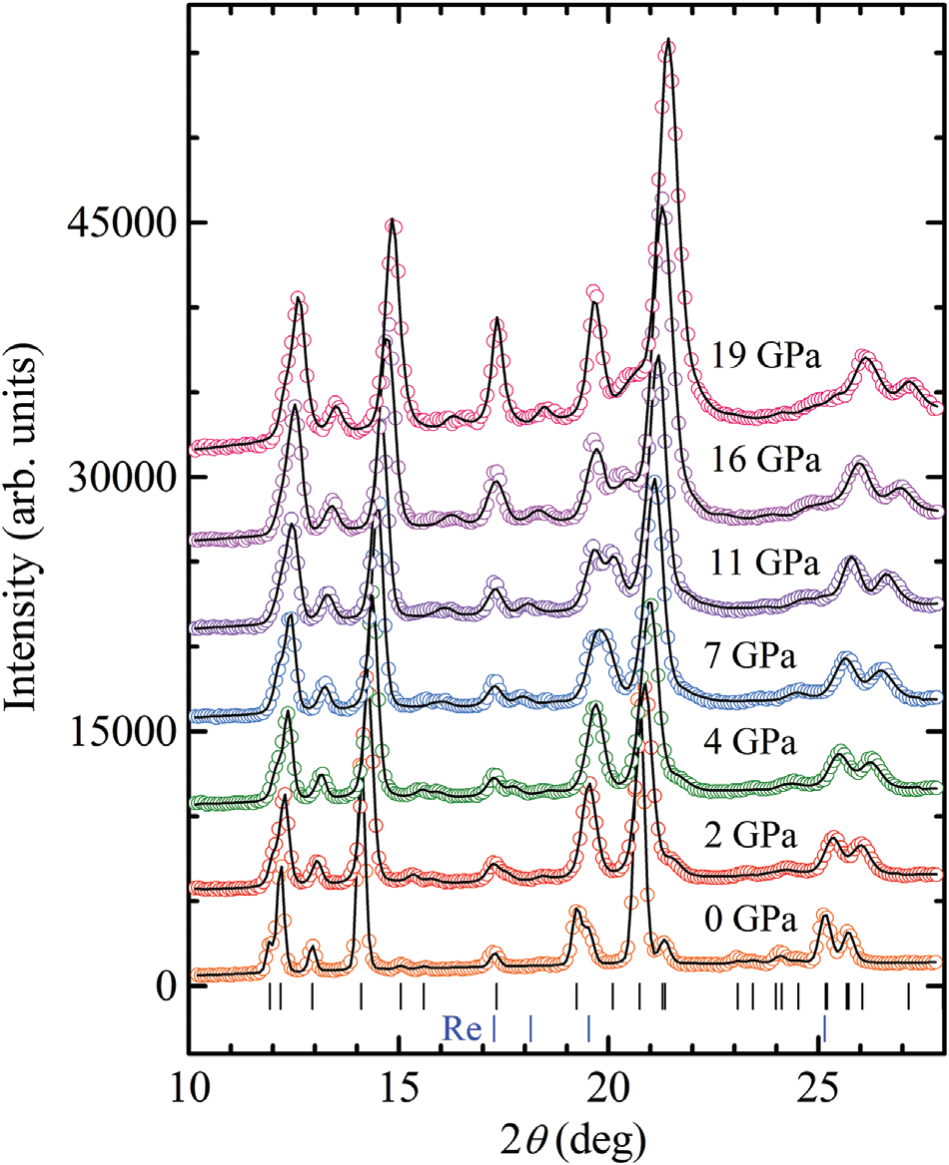
The X‐ray diffraction patterns of Fe_3_GeTe_2_ under various pressures, refined by the Rietveld method. The experimental points and calculated profiles are shown. The ticks below represent the calculated positions of the structural peaks of the hexagonal phase of Fe_3_GeTe_2_ and the additional diffraction peaks from the Re‐gasket.

The X‐ray diffraction patterns of Fe_3_GeTe_2_ under pressures up to 19 GPa are shown in **Figure** **4**. They are consistent with the hexagonal crystal structure of *P*6_3_/*mmc* symmetry. The values of the unit cell parameters obtained at ambient conditions, *a* = 3.9509(1) Å and *c* = 16.2855(2) Å, are consistent with the previous studies.^[^ ^35^, ^38^ ^]^ The structural parameters, determined at high pressures, are summarized in **Table** **1**. To characterize the XRD refinement quality, the *R* factors (*R_p_* and *R_wp_*) are also determined and included in this table. Since the determination uncertainties are small, the obtained structural parameters are accurate.

**Table 1:** The structural parameters of Fe_3_GeTe_2_ at selected pressures, obtained from XRD measurements. The atomic positions are: Fe1 – 4(e) (0, 0, *z*), Fe2 – 2(c) (1/3, 2/3, 1/4), Ge – 2(d) (1/3, 2/3, 3/4), Te – 4(f) (1/3, 2/3, *z*) of the space group *P6_3_/mmc*

*P*, GPa	0	2	4	7.3	11	16	19
*a*, Å	3.950(1)	3.930(1)	3.911(5)	3.887(3)	3.872(3)	3.851(1)	3.827(2)
*c*, Å	16.285(2)	15.998(3)	15.799(1)	15.500(3)	15.311(3)	15.028(1)	14.864(1)
Fe1: *z*	0.1757(5)	0.1739(8)	0.1733(8)	0.1731(8)	0.1738(9)	0.1750(9)	0.1765(9)
Te: *z*	0.0887(4)	0.0859(6)	0.0843(6)	0.0837(6)	0.0844(7)	0.0869(7)	0.0902(7)
*R_p_* (%)	6.51	5.89	5.99	5.69	6.78	7.84	7.70
*R_wp_* (%)	6.75	6.46	6.53	7.11	7.69	8.31	8.79

**Figure 5:**
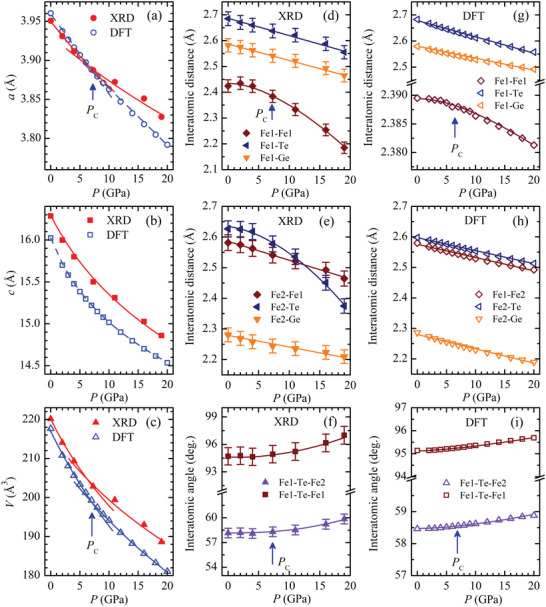
The unit cell parameters and volume of Fe_3_GeTe_2_ determined by XRD as functions of pressure, fitted by the third order Birch‐Murnaghan equation of state and results of DFT calculations (a–c). The determination errors do not exceed the symbols size. The interatomic distances and angles determined by XRD as functions of pressure (d–f) and results of DFT calculations (g–i). The lines are guides to eyes only. The bars represent the computational errors obtained by the Fullprof program.

The lattice contraction of Fe_3_GeTe_2_ is strongly anisotropic (**Figure** **5**). The significant average compressibility of the *c*‐axis, *k*_c_ = 0.0046 GPa^−1^, which is about twice larger than that of the *a*‐axis, is caused by the weak interlayer vdW bonds. The pressure behavior of the *a*‐axis demonstrates an anomaly at *P*_C_ ≈ 7 GPa (Figure 5). Below this pressure (*P* < *P*_C_), its average compressibility is *k_a_*  =  0.0021 GPa^−1^ and it is reduced substantially to *k_a_* = 0.0013 GPa^−1^ for *P* > *P*_C_. The unit cell volume under pressure also follows an unusual behavior for the *a*‐axis. Its bulk modulus and pressure derivative are evaluated to be *B*_0_ = 72(3) GPa and *B*′ = 5.0(5), from the third order Birch‐Murnaghan equation of state fitting in the pressure range *P* < *P*_C_ This changes to *B*_0_ = 92(4) GPa and *B*′ = 5.0 (fixed) for *P* > *P*_C_. The values obtained at moderate pressures are comparable with those reported for deficient Fe_3‐x_GeTe_2_,^[^ ^36^ ^]^ *B*_0_ = 52(8) GPa and *B*′ = 5.8(1), and a vdW transition metal dichalcogenide WSe_2_,^[^ ^46^ ^]^ *B*_0_ = 72(5) GPa and *B*′ = 4.6(5). The fact that the pressure anomaly observed at 7 GPa for the lattice parameter *a* but the lattice parameter *c* (Figure 5) indicates that this phenomenon is not due to effects of pressure gradient. Otherwise, the anomaly should appear on the pressure dependence of both parameters. We should also note here that while such structural change ≈7 GPa was not visible in the XRD spectra reported in work,^[^ ^38^ ^]^ it is visible in our XRD spectra (Figure 4) because of the higher resolution in the 2‐theta space.

The Fe(1,2)‐Ge(Te) and Fe1‐Fe2 bonds (Figure 5), are predominantly oriented within the (*ab*) planes of the layered units, which decrease linearly upon compression. In contrast, the out‐of‐plane Fe1‐Fe1 and Fe2‐Te bonds demonstrate a complex pressure behavior (Figure 5). In the pressure range below *P*_C_, they change quite weakly. For *P* > *P*_C_, these bonds are reduced much more rapidly in comparison to the in‐plane oriented bonds. The observed anomalous modification of these bonds may be caused by a competing character of in‐ plane and out‐of‐plane interatomic interactions. The latter ones increase more rapidly due to the significant reduction of the *c*‐axis and likely induce an isostructural phase transformation at *P* = *P*_C_, which is also visible as an anomaly in the pressure behavior of the *a*‐axis and unit cell volume. The modification of the interatomic Fe1‐Te‐Fe1 and Fe1‐Te‐Fe2 angles reflect the complex variation of the interatomic bonds and change weakly at *P* < *P*_C_. At higher pressures, their angles values increase nonlinearly (Figure 5).

### 2.3 Raman Spectroscopy

**Figure 6:**
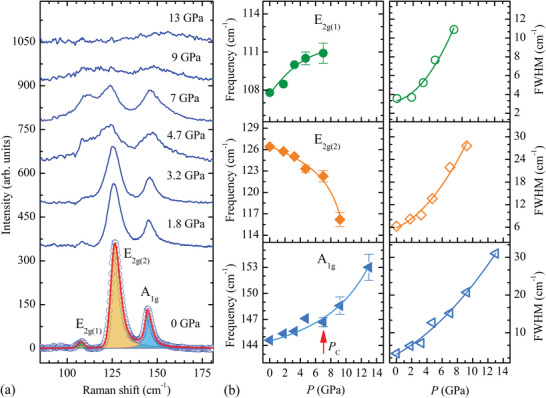
a) The Raman spectra of Fe_3_GeTe_2_ measured at *T* = 15 K under various pressures. The fitting of Raman spectrum at 0 GPa and 15 K to the pseudo‐Voigt function is shown. b) The pressure dependences of selected Raman modes frequencies and full width at half‐maximum (FWHM). The lines are guides to eyes only. The error bars below *P* = 9 GPa do not exceed the symbol sizes.

In the Raman spectra of Fe_3_GeTe_2_, measured at selected pressures and *T* = 15 K, three primary peaks at ≈108, 126, and 144 cm^−1^ (for *P* = 0 GPa) were observed (**Figure** **6**). They can be assigned to E_2g(1)_, E_2g(2)_ and A_1g_ modes, respectively.^[^ ^41^, ^44^ ^]^ The strongest E_2g(2)_ mode frequency demonstrates a pronounced pressure‐induced softening, which is enhanced in the vicinity of the critical pressure *P*_C_. This mode is associated with the in‐plane vibrations of Fe and Ge atoms with a partial contribution of the Te atoms.^[^ ^41^ ^]^ The A_1g_ mode frequency increases almost linearly at pressures below *P*_C_ and exhibits a rapid enlargement above *P*_C_. This mode is associated with the out‐of‐plane vibrations of Te atoms.^[^ ^41^ ^]^ The unusual pressure behavior of the strongest E_2g(2)_ and A_1g_ modes is likely related to the anomalous modification of Fe2‐Te distances (Figure 5) and also the Fe1‐Ge‐Fe1(2) interatomic angles. The weakest E_2g(1)_ mode exhibits a nearly linear increase at *P* < *P*_C_. It is difficult to determine its frequency at pressures above *P*_C_. All the observed modes are broadened significantly upon compression, increasing their linewidths by 4–5 times (Figure 6). This happens in the absence of the pronounced broadening of the diffraction peaks in the XRD data (Figure 3). Therefore, this effect is not related to loss of the long‐range atomic order but to the pressure‐induced modification of the electron‐phonon and spin‐phonon coupling, leading to a reduction in phonon lifetimes. A similar pressure behavior of the vibrational modes was also observed at ambient temperature above *T*_C_,^[^ ^44^ ^]^ implying that the structural modifications are likely the main driving mechanism for the observed phenomena.

### 2.4 DFT Calculations

To complement the experimental findings, DFT calculations have been performed. The structural parameters of Fe_3_GeTe_2_ at ambient pressure were calculated using different functionals, including LDA, LDA‐B86b, LDA‐FM, PBE, PBE‐D3, PBE‐B86b, PBE‐FM, and PBE‐D3‐FM. The LDA‐FM functional produces the most consistent results with the experimental parameters determined by XRD (**Table** **2**) and it was selected for further calculations at high pressures. The calculated high‐pressure dependences of lattice parameters and unit cell volume (Figure 5) qualitatively reproduces anomalies at *P*_C_ ≈ 7 GPa, manifested by the XRD measurements and associated with the structural transformation. However, the DFT results overestimate the lattice compression of the *a*‐axis in comparison to the XRD findings. Due to this reason, the calculated bulk modulus, and its pressure derivative, *B*_0‐DFT_ = 63 GPa and *B*′ = 5.0 in the pressure range below *P*_C_, and *B*_0‐DFT_ = 73 GPa and *B*′ = 5.0 for *P* > *P*_C_, are smaller than those determined from the experiments. The complex nonlinear pressure behaviors of the interatomic Fe1‐Fe1 bonds and Fe1‐Te‐Fe1, Fe1‐Te‐Fe2 angles in the vicinity of *P*_C_ are also reproduced qualitatively, although their relative variation is not so pronounced with respect to experimentally determined dependencies (Figure 5). The linear character of the pressure dependences of the Fe1(2)‐Te(Ge) bonds, found from the DFT calculations, is also in qualitative agreement with the experimental XRD results. DFT calculations also provide a linear pressure behavior of the Fe1‐Fe2 distance with a moderate compressibility, whereas the experimental data reveal more pronounced non‐linear compression of this bond (Figure 5). This is correlated with a rapid enlargement of the A_1g_ vibrational mode frequency above *P*_C_ (Figure 6). The mentioned differences in the experimental and theoretical pressure behavior of the structural parameters may be related to a gradual suppression of the FM state and increased itinerancy of the system.

**Table 2:** Structural parameters of Fe_3_GeTe_2_, obtained at ambient pressure by different calculation approaches

	XRD	LDA	LDA‐B86b	LDA‐FM	PBE	PBE‐D3	PBE‐B86b	PBE‐FM	PBE‐D3‐FM
*a* (Å)	3.950(1)	3.832	3.900	3.960	3.874	3.855	3.905	4.167	4.015
*c* (Å)	16.29(1)	15.711	16.240	16.022	15.814	15.511	15.995	15.906	15.677
*V* (Å^3^)	220.1(2)	199.79	213.935	217.633	205.493	199.636	211.217	239.194	218.919
Fe1: *x*	0	0	0	0	0	0	0	0	0
*y*	0	0	0	0	0	0	0	0	0
*z*	0.1757(4)	0.1736	0.1747	0.1754	0.1732	0.1718	0.1732	0.1701	0.1734
Fe2: *x*	1/3	1/3	1/3	1/3	1/3	1/3	1/3	1/3	1/3
*y*	2/3	2/3	2/3	2/3	2/3	2/3	2/3	2/3	2/3
*z*	1/4	1/4	1/4	1/4	1/4	1/4	1/4	1/4	1/4
Ge: *x*	1/3	1/3	1/3	1/3	1/3	1/3	1/3	1/3	1/3
*y*	2/3	2/3	2/3	2/3	2/3	2/3	2/3	2/3	2/3
*z*	0.0887(3)	0.0915	0.0935	0.0878	0.0918	0.0893	0.0916	0.0849	0.0828
Te: *x*	1/3	1/3	1/3	1/3	1/3	1/3	1/3	1/3	1/3
*y*	2/3	2/3	2/3	2/3	2/3	2/3	2/3	2/3	2/3
*z*	3/4	3/4	3/4	3/4	3/4	3/4	3/4	3/4	3/4

**Figure 7:**
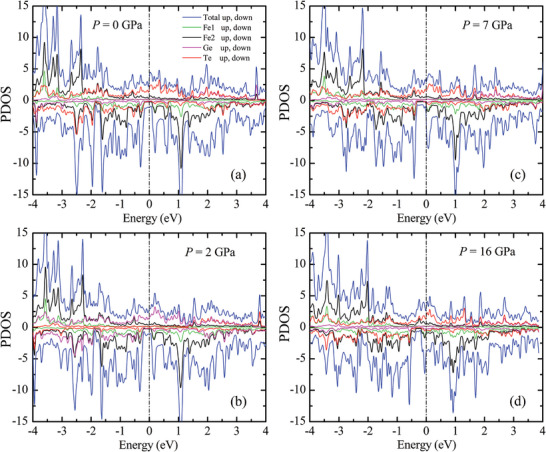
Total and partial densities of states in Fe_3_GeTe_2_, calculated for selected pressures of a) 0, b) 2, c) 7 and d) 16 GPa.

**Figure 8:**
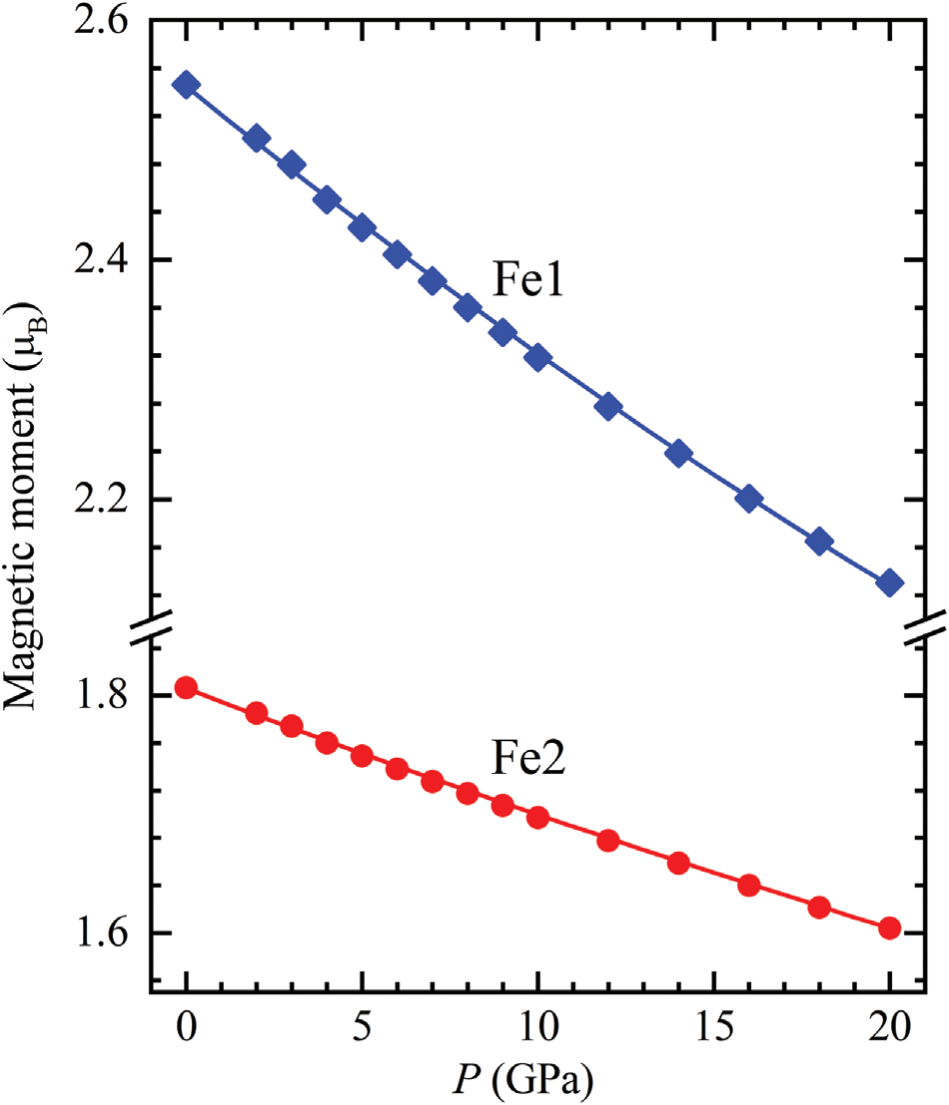
The pressure evolution of the Fe1 and Fe2 ordered magnetic moments, obtained from DFT calculations.

**Figure** **7** shows the density of states (DOS) at ambient pressure, wherein the Fermi energy (*E*_F_) is located in the vicinity of the pronounced peak in the spin‐down sub‐band. This sub‐band is formed by major contributions from Fe1,2 atoms and a small portion from Te atoms. Upon lattice compression, this peak shifts toward the *E*_F_ level, leading to substantial increase of the spin‐down DOS at *E*_F_ (Figure 7). The relevant electronic structure modifications lead to a rapid pressure‐induced suppression of the ordered magnetic moment, more prominent for the Fe1 atoms (**Figure** **8**).

It should be noted, that less pronounced tendency toward suppression of the local magnetic Fe moments was also found in previous DFT calculations,^[^ ^37^ ^]^ based on application of the SCAN method realized in VASP code. These calculations also point to a possible structural phase transformation at *P* ≈ 6 GPa, although the predicted pressure dependencies of interatomic distances are quite distinct from the experimental curves.

While the DFT calculations prove suppression of the ordered magnetic moments in the FM state, their values remain finite at pressures up to 20 GPa, in contrast to the experimentally observed total suppression of the ferromagnetic order and stabilization of a paramagnetic state above 14 GPa. This points to the restricted applicability of the LDA‐FM approach in the conditions of pressure induced enhancement of the system itinerancy. Further theoretical calculations by alternative methods like DMFT might be helpful to understand the features of a pressure‐induced ferromagnetic – paramagnetic crossover in Fe_3_GeTe_2_.

From the above experimental and theoretical findings, we now discuss the origin of the ferromagnetic suppression and the emergence of the paramagnetic state in Fe_3_GeTe_2_ under high pressures. We recall that the magnetism of Fe_3_GeTe_2_ is governed by a delicate balance of the direct exchange and superexchange interactions, which depend on the Fe‐Fe distances and Fe‐Te(Ge)‐Fe bond angles.^[^ ^34^, ^47^ ^]^ Since the *d*‐orbitals on the nearest‐neighbor Fe atoms overlap directly without a mediation atom, the direct exchange interaction among the Fe moments favors antiferromagnetic (AFM) coupling. When the Fe‐Fe (Fe1‐Fe1, Fe1‐Fe2) distances (or bond lengths) are decreased (upon compressive strain or under pressure), the AFM couplings are strengthened. Otherwise, the AFM couplings become weaker when the Fe‐Fe distances are increased (upon tensile strain). The superexchange FM couplings between Fe moments are mediated by Te or Ge atoms, depending on the Fe‐Te(Ge)‐Fe bond angles. These FM couplings become weaker or stronger when the Fe‐Te(Ge)‐Fe bond angles are increased (upon compressive strain or under pressure) or decreased (upon tensive strain). Accordingly, in our present case, the application of high pressures (compressive strain) to Fe_3_GeTe_2_ decreased the Fe1‐Fe1 and Fe1‐Fe2 distances (Figures 5d,e) and increased the Fe1‐Te(Ge)‐Fe1 and Fe1‐Te(Ge)‐Fe2 bond angles (Figure 5f). As a result, the FM couplings were weakened, and the AFM couplings were strengthened in the pressured Fe_3_GeTe_2_. It is worth noticing in Figure 5f that the Fe1‐Te(Ge)‐Fe1 and Fe1‐Te(Ge)‐Fe2 bond angles increased slowly in the pressure range *P* < *P*_C_ ≈ 7 GPa, but much more strongly for higher pressures (*P* > 7 GPa). A noticeable slope change in the dependence of the Fe1‐Fe1 distance on pressure was also observed ≈7 PGa (Figure 5d). Therefore, we argue that the unusual change in shape of the magnetic hysteresis loops observed experimentally ≈7 GPa by Wang et al.^[^ ^37^ ^]^ could be due to the combined changes in both the Fe1‐Fe1 distance and the Fe1‐Te(Ge)‐Fe1 and Fe1‐Te(Ge)‐Fe2 bond angles. While a full suppression of the FM state due to application of high pressures was not observed in Fe_3_GeTe_2_ by H. Wang et al.^[^ ^37^ ^]^ and X. Wang et al.,^[^ ^38^ ^]^ our synchrotron Mössbauer spectra analysis confirms this has happened ≈15 GPa, denoted as *P*_PM_ (Figure 3d). Values of *P*_PM_ could vary due to Fe deficiency in Fe_3‐_ *_x_* GeTe_2_.^[^ ^36^, ^37^, ^38^ ^]^ Our study not only helps resolve the discrepancy noticed above in the pressure‐induced structural change in Fe_3_GeTe_2_,^[^ ^36^, ^37^, ^38^ ^]^ but provides new insights into the underlying mechanism of strain‐mediated magnetism in other vdW magnetic systems.^[^ ^48^, ^49^, ^50^, ^51^ ^]^ Our study also suggests that Fe_3_GeTe_2_ is a perspective candidate material in the search for a pressure‐induced ferromagnetic quantum critical point.

## 3 Conclusions

In summary, we have demonstrated a pronounced high‐pressure response of magnetic and structural properties of the vdW itinerant ferromagnet Fe_3_GeTe_2_. The initial intrinsic FM state is suppressed gradually upon increased pressure, and there exists a critical pressure value (*P*_CM_ ≈ 15 GPa) above which only the paramagnetic state emerges. This finding implies that Fe_3_GeTe_2_ is a prospective candidate material in the search for a pressure‐induced ferromagnetic quantum critical point. The isostructural phase transformation at *P*_C_ ≈ 7 GPa is associated with the anomalous pressure behavior of the structural parameters. Interatomic distances, angles, and vibrational modes are driven by the competing character of in‐plane and out‐of‐plane interatomic interactions. DFT calculations performed using the LDA‐FM approach, qualitatively confirm the anomalous behavior of the structural parameters, affirming the experimentally observed isostructural phase transformation and its correlation with the magnetic change, as well as the gradual suppression of the FM state at high pressures.

## 4 Experimental Section

### Samples

The single crystalline Fe_3_GeTe_2_ samples were supplied by HQ Graphene.

### X‐Ray Diffraction

The X‐ray diffraction (XRD) measurements with powdered samples at high pressures up to 20 GPa and ambient temperature were performed with a Xeuss 3.0 instrument (Xenocs, France), equipped with the GeniX^3D^ source (Mo‐K_*α*_ edge, *λ* = 0.71078 Å) and Eiger 2R 500K detector (Dectris). The Almax Plate type diamond anvil cell (DAC, culets of 250 µm) without a pressure transmitting medium was used in the experiments. This is because the layered vdW Fe_3_GeTe_2_ was rather soft and its bulk moduli was small, which ensures nearly hydrostatic pressure distribution in the pressure cell. Furthermore, according to our previous experience, some composite compounds based on chemical reactions between the vdW materials and transmitting medium substance (gaseous like He, Ne, or liquid like methanol: ethanol mixture) was also formed if this transmitting medium substance was used. In our XRD experiments, the sample was loaded into a hole of 150 µm diameter made in a rhenium gasket intended to ≈50 µm thickness. The pressure was measured utilizing the ruby fluorescence technique. The exposure time for each pressure point was 2 h. The 2D XRD datasets were converted to the 1D diffraction patterns using the FIT2D program.^[^ ^52^ ^]^ The X‐ray diffraction patterns were analyzed by the Rietveld method using the Fullprof program.^[^ ^53^ ^]^

### Raman Spectroscopy

The Raman spectra at high pressures up to 13 GPa and temperature down to 15 K were collected using the LabRAM HR Evolution spectrometer (Horiba, France) with a wavelength excitation of 632.8 nm emitted from a He – Ne laser, 1800 grating, confocal hole of 200 µm, and x20 objective. A low vibration helium refrigerator (Advanced Research Systems, USA) was used for cooling of the DAC.

### Synchrotron Mössbauer Source Spectroscopy

Synchrotron Mössbauer source (SMS) spectroscopy measurements with a single crystalline sample were performed at the Nuclear Resonance beamline^[^ ^54^ ^]^ ID18 at the European Synchrotron Radiation Facility (ESRF) using the setup described.^[^ ^55^ ^]^ Experiments were carried out from ambient pressure up to 20 GPa, and in the temperature range between 10 – 290 K. The size of the x‐ray beam spot at the sample was ≈12 µm and 4 µm in vertical and horizontal directions, respectively. The BETSA‐type membrane diamond anvil cell (DAC) available at ESRF with diamond culets of 250 µm was used. The sample was loaded into a Re gasket indented to ≈30 µm thickness with an initial hole diameter of 150 µm. The pressure was determined by the ruby fluorescence technique using the Dewaele calibration scale.^[^ ^56^ ^]^ A He flow cryostat was used for low temperature measurements. The SMS data were fitted with the MossA software^[^ ^57^ ^]^ to obtain hyperfine parameters.

### DFT Calculations

DFT calculations were performed using projector augmented‐wave (PAW) pseudopotentials in Quantum‐ESPRESSO.^[^ ^58^, ^59^ ^]^ In order to choose a suitable exchange‐correlation functional to describe the physical properties of Fe_3_GeTe_2_, several ambient‐pressure structural calculations were performed by investigating the paramagnetic and FM configurations using the local density approximation (LDA), Perdew‐Burke‐Ernzerhof (PBE) exchange‐correlation functionals.^[^ ^60^ ^]^ The optB86b‐vdW (B86b) and DFT‐D3 (D3) functionals were applied for the vdW correction.^[^ ^61^, ^62^ ^]^ The LDA functional was selected for further calculations at high pressures. The electronic wave function was expanded in a plane wave basis set with an energy cutoff of 80 Ry. The charge density was expanded in a basis set with a plane wave cutoff of 800 Ry. A 15 × 15 × 4 Monkhorst‐Pack grid is used for the Brillouin zone sampling.^[^ ^63^ ^]^ All ionic relaxations and cell optimizations were performed using a threshold of 10^−5^ Ry/Bohr and 10^−6^ Ry for corresponding the force and energy.

## Conflict of Interest

The authors declare no conflict of interest.

## Author Contributions

D.P.K. initiated the research. S.E.K., D.P.K., and E.V.L. participated in the SMS experiments and performed data analysis. O.N.L. and E.V.L. performed the X‐ray diffraction experiments and data analysis. O.N.L. and N.O.G. performed the Raman spectroscopy experiments and data analysis. N.T.D., M.H.P., D.L.D., T.L.P., and T.A.T. performed the DFT calculations. D.P.K., O.N.L., N.T.D., and M.H.P. prepared the first draft of the manuscript and all the co‐authors contributed to the final version of the manuscript.

## Data Availability

Research data are not shared.
